# A two-gene strategy increases iron and zinc concentrations in wheat flour, improving mineral bioaccessibility

**DOI:** 10.1093/plphys/kiac499

**Published:** 2022-10-29

**Authors:** Sophie A Harrington, James M Connorton, Natasha I M Nyangoma, Rose McNelly, Yvie M L Morgan, Mohamad F Aslam, Paul A Sharp, Alexander A T Johnson, Cristobal Uauy, Janneke Balk

**Affiliations:** John Innes Centre, Norwich Research Park, Norwich NR4 7UH, UK; John Innes Centre, Norwich Research Park, Norwich NR4 7UH, UK; School of Biological Sciences, University of East Anglia, Norwich NR4 7TJ, UK; School of BioSciences, The University of Melbourne, Victoria 3010, Australia; John Innes Centre, Norwich Research Park, Norwich NR4 7UH, UK; School of Biological Sciences, University of East Anglia, Norwich NR4 7TJ, UK; John Innes Centre, Norwich Research Park, Norwich NR4 7UH, UK; School of Biological Sciences, University of East Anglia, Norwich NR4 7TJ, UK; Department of Nutritional Sciences, King’s College London, London SE1 9NH, UK; Department of Nutritional Sciences, King’s College London, London SE1 9NH, UK; School of BioSciences, The University of Melbourne, Victoria 3010, Australia; John Innes Centre, Norwich Research Park, Norwich NR4 7UH, UK; John Innes Centre, Norwich Research Park, Norwich NR4 7UH, UK; School of Biological Sciences, University of East Anglia, Norwich NR4 7TJ, UK

## Abstract

Dietary deficiencies of iron and zinc cause human malnutrition that can be mitigated by biofortified staple crops. Conventional breeding approaches to increase grain mineral concentrations in wheat (*Triticum aestivum* L.) have had only limited success, and our understanding of the genetic and physiological barriers to altering this trait is incomplete. Here we demonstrate that a transgenic approach combining endosperm-specific expression of the wheat *VACUOLAR IRON TRANSPORTER* gene *TaVIT2-D* with constitutive expression of the rice (*Oryza sativa*) *NICOTIANAMINE SYNTHASE* gene *OsNAS2* significantly increases the total concentration of zinc and relocates iron to white-flour fractions. In two distinct bread wheat cultivars, we show that the so called VIT-NAS construct led to a two-fold increase in zinc in wholemeal flour, to ∼50 µg g^−1^. Total iron was not significantly increased, but redistribution within the grain resulted in a three-fold increase in iron in highly pure, roller-milled white flour, to ∼25 µg g^−1^. Interestingly, expression of *OsNAS2* partially restored iron translocation to the aleurone, which is iron depleted in grain overexpressing *TaVIT2* alone. A greater than three-fold increase in the level of the natural plant metal chelator nicotianamine in the grain of VIT-NAS lines corresponded with improved iron and zinc bioaccessibility in white flour. The growth of VIT-NAS plants in the greenhouse was indistinguishable from untransformed controls. Our results provide insights into mineral translocation and distribution in wheat grain and demonstrate that the individual and combined effects of the two transgenes can enhance the nutritional quality of wheat beyond what is possible by conventional breeding.

## Teaser

Targeted expression of a vacuolar iron transporter and increased nicotianamine levels have combinatorial effects on iron and zinc levels and their distribution in wheat grain.

## Introduction

Low dietary intake of the essential mineral micronutrients iron and zinc from staple crops contributes to the global burden of malnutrition. Iron deficiency is the leading cause of anemia worldwide, particularly affecting young children and adult females (WHO, 2020). In children, iron deficiency can lead to stunted development, while in adults it can severely hinder economic productivity and greatly enhances the risk of maternal death in childbirth (WHO, 2015). Zinc deficiency affects all age groups and genders, manifested in stunting and reduced immunity to infectious diseases (Ritchie and Roser, 2017). In some South Asian and sub-Saharan countries with cereal-dominated diets, over half of the population is predicted to be zinc deficient (Kumssa et al., 2015).

Bread wheat (*Triticum aestivum* L.) is a global staple that provides between 20% and 25% of calories worldwide (FAO, 2021). Yet a combination of uneven nutrient distribution and associated anti-nutrient factors within the grain make wheat a suboptimal source of iron and zinc in human diets (Balk et al., 2019). Iron and zinc are predominantly located in the aleurone tissue and embryo (O'Dell et al., 1972; Ozturk et al., 2006; Cakmak et al., 2010; Moore et al., 2012). These parts of the grain are removed during industrial-scale roller milling of white flour, leading to the loss of 65%–75% of the iron and zinc present in the whole grain (Tang et al., 2008; Balk et al., 2019). Additionally, the bran fractions enriched in aleurone, pericarp, and embryo tissues contain high amounts of phytate (myoinositol-1,2,3,4,5,6-hexakisphosphate), an anti-nutrient that inhibits iron and zinc bioavailability (Hurrell and Egli, 2010; Gibson et al., 2018). Efforts to improve iron and zinc concentrations in wheat, therefore, must deal with the complementary issues of low levels of mineral micronutrients in the starchy endosperm, and low bioavailability in the outer cell layers comprising the bran.

Attempts have been made to leverage natural variation in wheat germplasm to increase grain iron and zinc concentrations by conventional breeding. A recent analysis of mineral micronutrients in the Watkins panel of wheat landraces identified variation of 24–49 µg g^−1^ zinc in the wholemeal flour, and 8–15 µg g^−1^ zinc in white flour (Khokhar et al., 2020). Similar levels of variation in zinc have been found in panels of Indian-adapted wheat varieties and CIMMYT germplasm (Velu et al., 2014; Khokhar et al., 2018; Velu et al., 2018). Variation in iron concentrations is also seen, particularly in wheat landraces and wild relatives; a panel of 170 elite, landrace, and wild relative varieties had wholemeal iron concentrations ranging from 25 to 56 µg g^−1^ iron (Monasterio and Graham, 2000). This existing genetic variation has been used to improve wholegrain micronutrient levels in elite wheat varieties. For example, introgression of the transcription factor *NO APICAL MERISTEM* (*NAM-B1*) from wild emmer wheat (*Triticum turgidum* ssp. *dicoccoides*) into bread wheat led to an increase in iron and zinc content of 18% and 12%, respectively (Uauy et al., 2006; Distelfeld et al., 2007). However, while genetic variation in the wholegrain iron concentration exists within germplasm stocks, to our knowledge no significant natural variation has been observed for iron in white flour, nor for increased iron bioavailability (Zhao et al., 2009). As a result, research effort has turned toward implementing transgenic and cisgenic approaches to improve iron and zinc levels and their bioavailability (Borrill et al., 2014; Balk et al., 2019).

Early work using transgenes to enhance micronutrient levels was carried out in rice (*Oryza sativa*), exploiting genes involved in iron uptake, transport, and storage. *NICOTIANAMINE SYNTHASE* (*NAS*), encoding the enzyme that synthesizes the metal chelator nicotianamine (NA), plays a key role in iron uptake and mobility of divalent metal ions in plants (Laffont and Arnoux, 2020). Overexpression of either of the three individual *NAS* genes in rice not only led to increased concentrations of NA, iron, and zinc in rice grains, but also improved iron bioavailability (Lee et al., 2009, 2011; Usuda et al., 2009; Johnson et al., 2011). An alternative approach to improving grain micronutrient content, later combined with *NAS* overexpression, focused on the iron storage protein ferritin. Endosperm-specific expression of the soybean (*Glycine max*) *FERRITIN* gene in rice led to an increase in the iron concentration in polished grains by as much as three-fold (Goto et al., 1999; Qu et al., 2005).

Initial efforts to biofortify wheat flour with iron and zinc built on the findings in rice, targeting similar candidate genes. Endosperm-specific expression of a *FERRITIN* gene from wheat or bean (*Phaseolus vulgaris*) in bread wheat led to an increase of ∼60% in total grain iron (Borg, 2012; Singh et al., 2017); however, X-Ray Fluorescence imaging showed that iron accumulated in the crease of the grain, not in the starchy endosperm (Neal et al., 2013), and would thus be removed by milling. High expression of the rice *OsNAS2* gene in wheat under a constitutive promoter led to 40%–100% more iron and 60%–250% more zinc in whole grains (Singh et al., 2017). Field trials of wheat lines generated in a different study but with a similar *OsNAS2* gene cassette showed increases of up to 30% more iron and up to 50% more zinc in whole grain, although with considerable variation between years and different field locations. The lines showed a robust >200% increase in NA, and improved bioavailability of iron in bread baked from biofortified flour (Beasley et al., 2021). Another strategy to modify grain iron was demonstrated in wheat by overexpressing the wheat *VACUOLAR IRON TRANSPORTER 2* gene (*TaVIT2-D*) under the wheat endosperm-specific *HIGH MOLECULAR WEIGHT GLUTENIN* (*HMWG*)*-D1* promoter (Connorton et al., 2017; Sheraz et al., 2021). The benefit of this strategy is the striking redistribution of iron to the endosperm adjacent to the embryo, with a consistent ∼200% increase in iron in hand-milled white flour and ≥250% increase in highly pure, roller-milled white-flour fractions, from 8 to 20 µg g^−1^ iron (Connorton et al., 2017; Balk et al., 2019). This concentration of iron is within the range of mandatory fortification levels of cereal flours and matches biofortification targets (Bouis et al., 2011), but cannot be achieved by expressing *NAS* genes alone.

Here we report on the results of combining endosperm expression of the *TaVIT2-D* gene with constitutive expression of the *OsNAS2* gene in two genetically distinct bread wheat cultivars. The grain from the so-called VIT-NAS transgenic plants showed the combinatorial effects of each transgene, namely redistribution of iron to the endosperm and higher total zinc and NA, the latter corresponding with increased mineral bioaccessibility. Total grain iron was not increased in these lines, but *OsNAS2* expression helped to overcome impaired iron translocation to the aleurone. Thus, combining the two transgenes in one cassette provides nutritional benefits well beyond those achieved by breeding approaches. In addition, the data provide insights in grain loading and distribution of minerals in wheat grain.

## Results

### The VIT-NAS construct drives high levels of *TaVIT2* and *OsNAS2* expression in grain and leaf tissue

**Figure 1:**
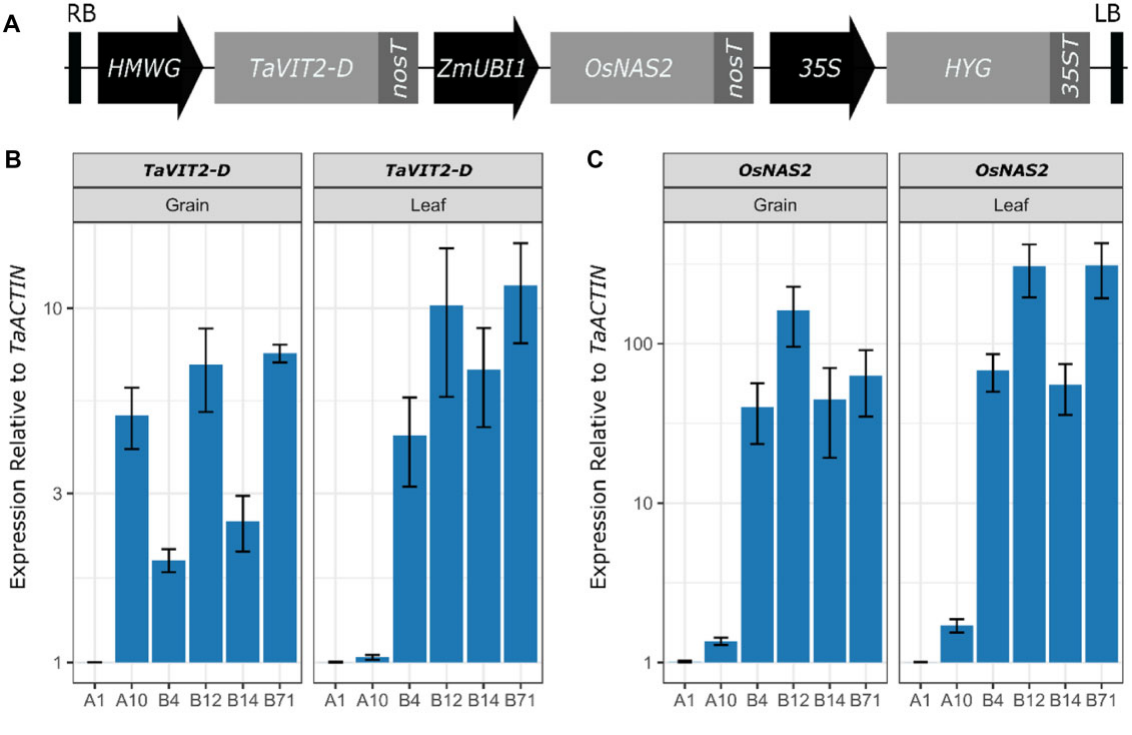
Expression of *TaVIT2-D* and *OsNAS2* in the VIT-NAS lines. A, Diagram of the T-DNA construct (not to scale): *HMWG*, *HIGH-MOLECULAR-WEIGHT GLUTENIN-D1-1* promoter; *TaVIT2-D*, wheat (*T. aestivum*) *VACUOLAR IRON TRANSPORTER2-D* gene; *nosT* (bacterial) *nopaline synthase* terminator; *ZmUBI1*, maize (*Zea mays*) *UBIQUITIN* promoter; *OsNAS2*, rice (*O. sativa*) *NICOTIANAMINE SYNTHASE 2* gene; *35S*, Cauliflower Mosaic Virus 35S promoter; *HYG*, hygromycin resistance gene; *35ST*, Cauliflower Mosaic Virus 35S terminator; LB, left border. B and C, RT–qPCR expression of (B) *TaVIT2-D* using primers specific for the transgene and (C) *OsNAS2* in grain (left) and flag leaf (right) tissue at 21 days postanthesis. Expression levels were calculated relative to *TaACTIN* for null transformant (A1) and transgenic (A10, B4, B12, B14, and B71) lines, and represented on a base-10 log scale. Error bars represent the standard error of three biological replicates for each line.

To increase the amount of iron and zinc in the wheat grain, while maximizing the concentration in the endosperm, we generated a construct, referred to as VIT-NAS, combining the *HMWG::TaVIT2-D* cassette (Connorton et al., 2017) with the *ZmUBI1::OsNAS2* cassette (Beasley et al., 2019) in a binary T-DNA vector with the hygromycin resistance marker (Figure 1A). Transformation of the VIT-NAS construct was independently carried out in both the cultivar Fielder, nowadays only used for research, and the cultivar Gladius, a drought-resistant variety grown in Australia. Following selection for hygromycin resistance, a total of 13 independent transformants were obtained in the cv. Fielder, with copy numbers ranging from one (in two transformants) to more than 12. Iron staining of grains was used to select five independent transformants alongside a null transformant for further characterization in homozygous lines of the T_3_ generation (Table 1). In cv. Gladius, 37 independent transformation events were obtained of which 3 independent lines with a single insert of the T-DNA—BD1-T, BD4-T, and BD7-T, alongside their null-segregant siblings—were selected for analysis in the T_1_ generation.

**Table 1:** VIT-NAS independent transformants in cv. Fielder and controls

Line	Copy Number (2n)
A1	0
A10	2
B4	4
B12	∼12^a^
B14	6
B71	>20^a^
TaVIT2^b^	8
22-15^b^	0

a The accuracy of the TaqMan-based copy number determination declines at higher numbers.

b TaVIT2 is line 22-19 as described in (Connorton et al., 2017) and 22-15 is a null transformant from the same study.

Expression analysis by reverse transcription–quantitative PCR (RT–qPCR) confirmed high levels of *TaVIT2-D* and *OsNAS2* expression in the cv. Fielder VIT-NAS lines compared to the null line A1 (Figure 1, B and C). Line A10 had very low expression of *OsNAS2* in both the grain and leaf tissue compared to the other VIT-NAS lines, indicating that the gene is not properly transcribed in this line (Figure 1C). The expression of the *TaVIT2-D* transgene in A10 is high in the grain and low in leaves, as expected from the endosperm-specific *HMWG* promoter (Figure 1B). Unexpectedly, the other VIT-NAS lines had elevated expression levels of the introduced *TaVIT2-D* gene in leaf tissue. We speculate this may be due to the insertion of several copies of the T-DNA in tandem, allowing the *ZmUBI1* or *35S* promoters to influence *TaVIT2-D* expression (see “Discussion”).

### Combined overexpression of *TaVIT2* and *OsNAS2* does not affect plant growth

**Figure 2:**
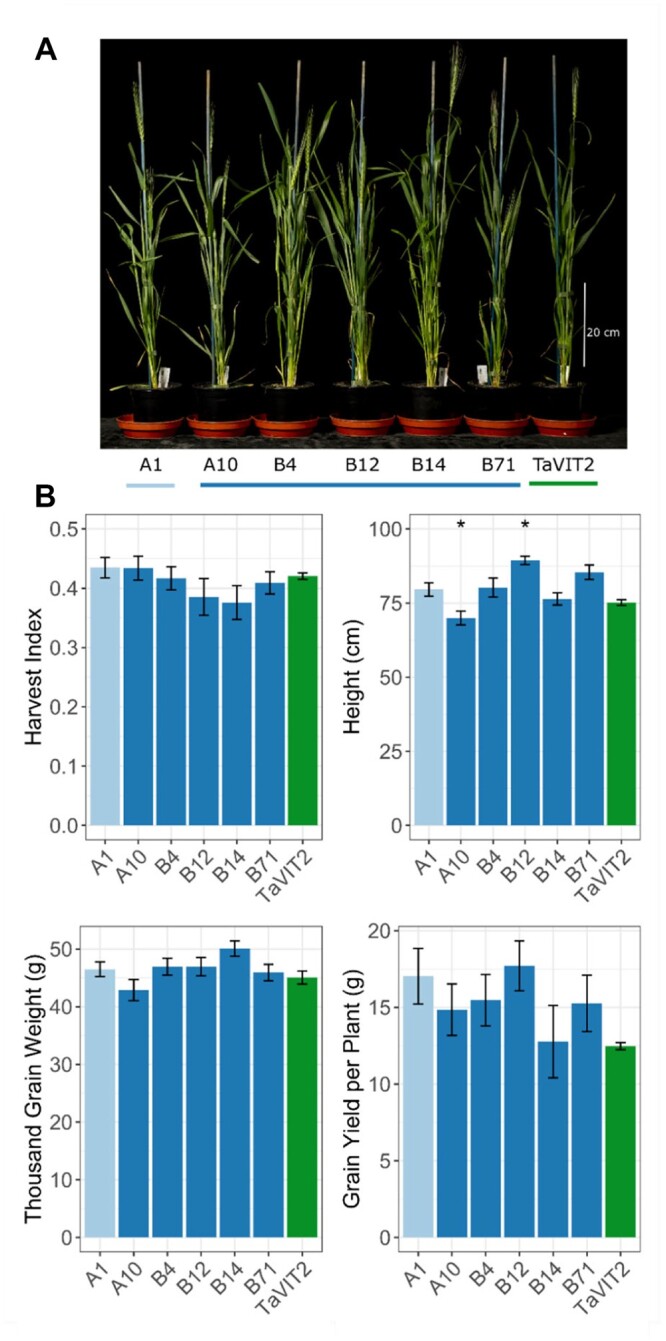
The VIT-NAS construct does not affect plant growth. A, Representative individual wheat plants of the VIT-NAS T_3_ generation at 5 days after anthesis. B, Plant growth parameters including harvest index, height, thousand grain weight, and grain yield per plant in the null transformant (A1; light blue), VIT-NAS (A10, B4, B12, B14, B71; dark blue), and TaVIT2 (green) lines (*P < 0.05, Dunnett’s test against A1). Error bars are the standard error of five biological replicates.

To investigate whether the expression of the VIT-NAS construct affects plant growth, we measured multiple growth and yield parameters for both the cv. Fielder (Figure 2; Supplemental Figure S1) and cv. Gladius transformants (Supplemental Figure S2). In general, no significant differences in plant growth were seen between the null transformant A1 and the VIT-NAS lines (Figure 2B; Supplemental Figures S1 and S2), nor any signs of either iron deficiency (chlorosis), zinc deficiency, or toxicity symptoms. Some individual lines had a small but significant difference in plant height, with cv. Fielder line A10 being shorter than the null segregant, while line B12 was significantly taller (*P* < 0.05, Dunnett Test against A1; Figure 2). A single transformant from cv. Gladius, BD1-T, had a lower tiller number than the corresponding null sibling line (*P* < 0.001; Supplemental Figure S2). The average grain area and length were significantly increased in B14 (*P* < 0.01 and 0.001, respectively; Dunnett’s test against A1; Supplemental Figure S1). The remaining lines and traits, including harvest index, grain yield per plant, and thousand grain weight, showed no significant difference against the controls (Figure 2B; Supplemental Figures S1 and S2).

### The VIT-NAS lines have increased concentrations of iron and zinc in white and wholemeal flours

**Figure 3:**
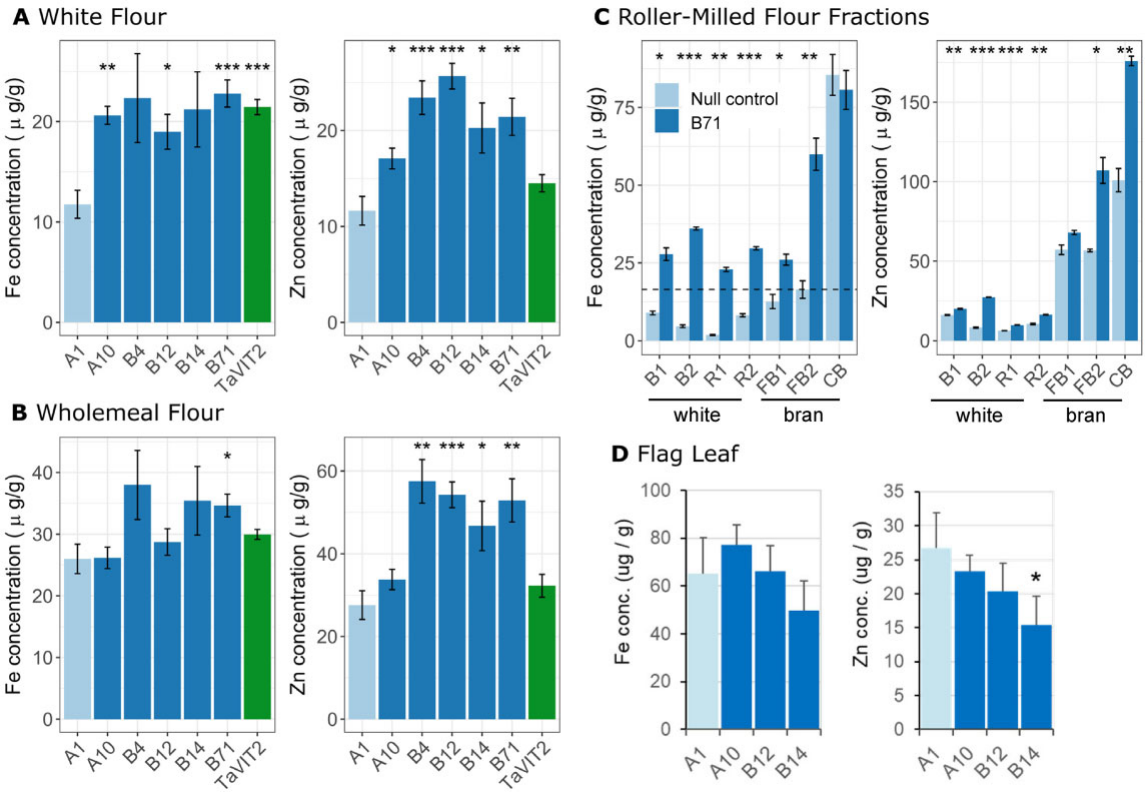
The VIT-NAS lines have increased iron and zinc concentrations in flour fractions. A and B, Iron and zinc concentrations measured by ICP-OES in white flour (A) and wholemeal flour (B) of the null transformant (A1, light blue), VIT-NAS (A10, B4, B12, B14, and B71; dark blue), and TaVIT2 (green) lines in the homozygous T_3_ generation. Error bars represent the standard error of five biological replicates. Student’s *t* test against the null (A1); *P < 0.05, **P < 0.01, ***P < 0.001. C, Iron and zinc concentrations in roller-milled fractions of grain from a null transformant (light blue) and VIT-NAS (B71, dark blue). The different flour fractions are: (white flour) B1, first break; B2, second break; R1, first reduction; R2, second reduction; (bran) FB1, first FB; FB2, second FB. The dashed line represents the minimum requirement for iron fortification in white flour in the UK (16.5 µg g^−1^). Error bars represent the standard error of three technical replicates. Student’s *t* test between null and B71 for each fraction; *P < 0.05, **P < 0.01, ***P < 0.001. D, Iron and zinc concentrations in flag leaf tissue 21-day postanthesis. Error bars represent the standard error of three to four biological replicates. Student’s *t* test against the null (A1); *P < 0.05.

To quantify the levels of iron and zinc in flours, grains were hand-milled to obtain wholemeal flour which was sieved to obtain a crude white-flour fraction. Concentrations of key micronutrients were measured using Inductively Coupled Plasma-Optical Emission Spectroscopy (ICP-OES). All VIT-NAS lines, in both cv. Fielder and cv. Gladius had approximately two-fold higher concentrations of iron in the white-flour fraction compared with the controls, which was statistically significant in all Gladius lines and in three of the five Fielder lines (*P* < 0.05, Student’s *t* test; Figure 3A; Supplemental Figure S3). The two-fold increase in iron in white flour is similar to that seen in lines transformed with *HMWG::TaVIT2* alone (Figure 3A and (Connorton et al., 2017)). In contrast to the TaVIT2 line, all cv. Fielder VIT-NAS lines had significantly higher concentrations of zinc in the white-flour fraction compared to the control lines (*P* < 0.05, Student’s *t* test; Figure 3A). Similarly, two of the three cv. Gladius VIT-NAS lines had significantly higher white-flour zinc concentrations (*P* < 0.05, Student’s *t* test; Supplemental Figure S3).

In wholemeal flour, only one VIT-NAS line, B71, had significantly more iron than the control (30% more, *P* < 0.05, Student’s *t* test; Figure 3B). Lines B4 and B14 also showed an increase of ∼30% more iron, but this was not significant. While the increase in wholemeal iron is less than seen previously in a study of *OsNAS2*-overexpressing wheat (Singh et al., 2017), it is similar to values seen in field studies (Beasley et al., 2021). Wholemeal zinc concentrations were increased approximately two-fold in all VIT-NAS lines except A10 (*P* < 0.05, Student’s *t* test; Figure 3B). As noted before, the expression level of *OsNAS2* in the A10 line is low (Figure 1B) and thus the A10 line is equivalent to the TaVIT2 line. Correspondingly, wholemeal zinc levels in both A10 and TaVIT2 are not significantly higher than in the control line. This further demonstrates the specific effect of *OsNAS2* overexpression on increasing the grain zinc concentration.

To obtain cleaner white-flour fractions, grain pooled from several plants of the VIT-NAS line B71 and a null transformant were milled using a laboratory-scale roller mill. Line B71 was chosen because it has the most significant increases in iron for both white and wholemeal flour. Iron and zinc concentrations are normally between 5 and 10 µg g^−1^ in Break (B) and Reduction (R) white-flour fractions in industrially milled grain (Balk et al., 2019), similar to the concentrations we measured in the corresponding white-flour fractions of the null transformant (Figure 3C). In the VIT-NAS lines, the iron concentration was significantly increased by three- to seven-fold, to 23–35 µg g^−1^, and was also significantly increased in the two Fine Bran (FB) fractions (*P* < 0.05, Student’s *t* test; Figure 3C). A less dramatic but significant 2.5-fold increase in zinc concentration was found in the white-flour fractions, as well as a two-fold increase in zinc in FB2 and coarse bran (CB). The increased zinc levels in VIT-NAS flour contrast with the previous analysis of the TaVIT2 line, which did not have significantly increased zinc in the roller-milled white-flour fractions (Balk et al., 2019). This emphasizes both the substrate specificity of the iron TaVIT2 transporter (Connorton et al., 2017) and the key role of *OsNAS2* expression in increasing zinc in wheat flours.

Because of the unexpected high expression of the *TaVIT2-D* transgene in flag leaves of plants harboring multiple inserts of the T-DNA (Figure 1B), we measured iron and zinc in this tissue by ICP-OES. Flag leaves were sampled 21 days after anthesis, at the same time point as for RT–qPCR analysis. The iron and zinc concentrations of ∼60 µg g^−1^ and 25 µg g^−1^, respectively, in the null transformant (A1) are typical for leaves. The flag leaves of B12 and B14 contained a similar concentration of iron as A1, despite high *TaVIT2-D* expression, and there was slightly less zinc in B14 leaves (Figure 3D).

### The distribution of iron and zinc within the grain is influenced by both the *TaVIT2* and *OsNAS2* transgenes

**Figure 4:**
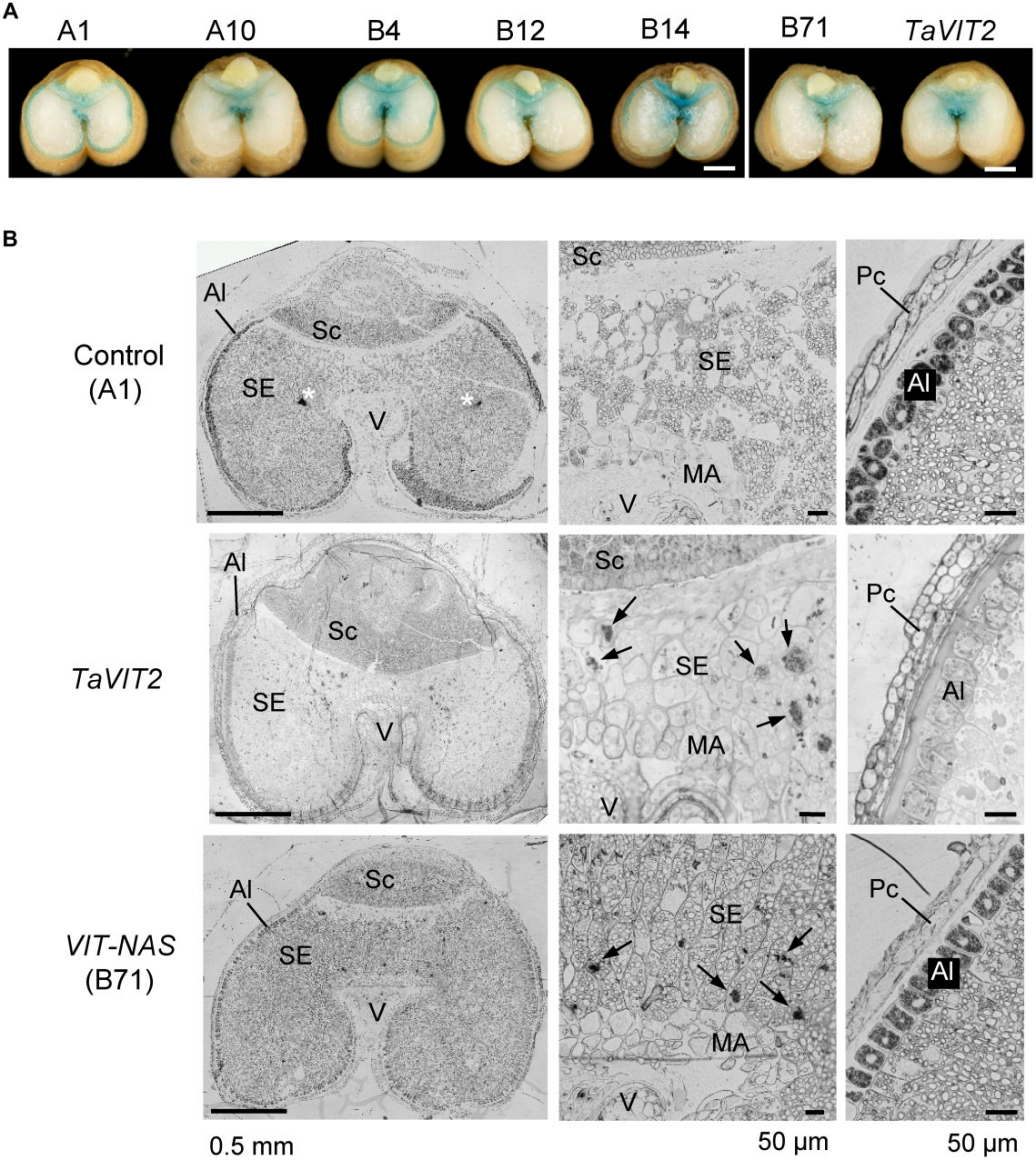
*TaVIT2* and *OsNAS2* affect the distribution of iron in the grain. A, Cross sections of mature grains stained for iron (blue) using the Perls’ method. Scale bars are 1 mm. B, Thin sections (1 µm) of immature grains 21 days after anthesis stained for iron (black in monochrome images) using the Perls’-diaminobenzidine method. Left, cross-section through the grain; middle, detail at higher magnification of the starchy endosperm between the vascular bundle and embryo; right, detail including the aleurone tissue. The images are representative of two grains taken from two different plants from the indicated wheat lines. Perls’-diaminobenziden staining of TaVIT2 grain was previously described (Sheraz et al., 2021). Al, aleurone; MA, modified aleurone; Pc, pericarp; Sc, scutellum of the embryo; SE, starchy endosperm; V, vascular bundle (maternal tissue). White asterisk indicates a nonspecific dye precipitate; black arrows point at iron accumulation in the vacuoles of starchy endosperm cells. Scale bars as indicated.

The dramatic increase in iron concentration in hand-milled and roller-milled white flours from VIT-NAS lines suggested that iron accumulates in the starchy endosperm. To further investigate the spatial distribution of iron within the grain, we carried out Perls’ staining on cross-sections of mature grains. Similar to the TaVIT2 line (Sheraz et al., 2021), grains from VIT-NAS lines accumulated iron in the central region of the endosperm, between the maternal vascular bundle and the embryo (Figure 4, A and B). Iron accumulation in the endosperm of TaVIT2 lines is correlated with decreased iron in the aleurone tissue and embryo (Sheraz et al., 2021). For the aleurone, this can be made visual by Perls’ staining in whole grain cross-sections as a lack of a blue outline (compare A1 and TaVIT2 in Figure 4A). In contrast, the VIT-NAS grains from lines B4, B12, B14, and B71, do show iron staining in the aleurone tissue, although variation of staining intensity suggests that the iron concentration may be less than in control grains.

We also carried out Perls’ staining enhanced with diaminobenzidine on thin sections of immature grains from line B71, alongside the TaVIT2 line and the null control. Iron accumulation in globoid clusters was visible in central starchy endosperm cells in both the VIT-NAS and TaVIT2 lines, but not in the control line. Moreover, iron was visibly retained in the aleurone tissue in the B71 line, compared to near-depletion in the TaVIT2 line (Figure 4B).

### VIT-NAS flour has increased iron and zinc bioaccessibility

**Figure 5:**
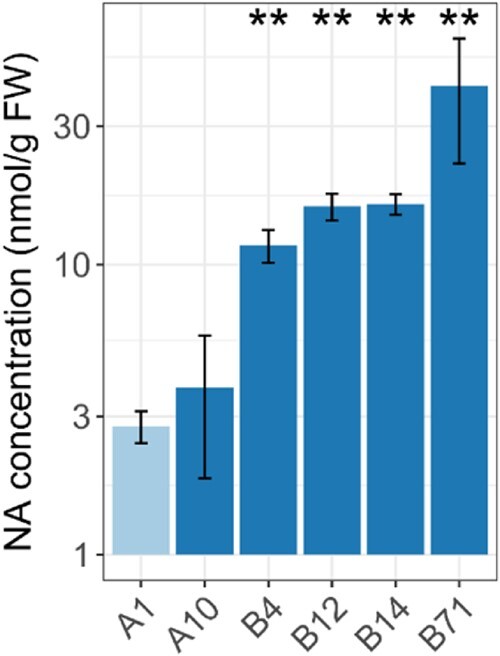
The VIT-NAS lines contain higher levels of NA. NA concentration in grain of the control (A1, light blue) and VIT-NAS (A10, B4, B12, B14, and B71, dark blue) lines. NA levels are plotted against a base-10 log scale. Error bars represent the standard error of three biological replicates. **P < 0.01; Mann–Whitney test compared to the control line.

To measure the extent to which overexpression of *OsNAS2* increases the concentration of NA, we carried out High Pressure Liquid Chromatography-Mass Spectrometry (HPLC-MS) on wholemeal flour samples (Banakar et al., 2017). We saw significant increases in NA between three- and ten-fold above control (*P* < 0.01, Mann–Whitney test; Figure 5). The wheat line without significantly increased NA levels, A10, was also the line that had failed to properly induce *OsNAS2* expression (Figure 1B). The fold increase in NA is similar to that previously reported in grain of lines overexpressing *OsNAS2* alone, which were single-copy insertion lines (Beasley et al., 2019, 2021). Lines with two or more T-DNA inserts, and thus more copies of *OsNAS2*, displayed only a weak trend for higher grain NA concentrations. The concentration of deoxymugineic acid (DMA), a downstream metabolite of NA also associated with mineral bioavailability, was not measured; however, DMA is expected to be ∼70% of the NA levels, as shown by extensive analysis in OsNAS2-expressing wheat (Beasley et al., 2021).

We also measured the amount of phytate, an anti-nutrient that inhibits iron and zinc bioavailability, in white and wholemeal flour fractions. We observed no significant differences in phytate concentrations in the VIT-NAS lines compared to the control line. The low concentration of phytate in white flour resulted in a two-fold increase in the iron-to-phytate molar ratio for three of the five lines tested, similar to the TaVIT2 line (Supplemental Figure S4; *P* < 0.05, Student’s *t* test).

We then investigated the bioaccessibility of the iron and zinc levels in white flour from the VIT-NAS line. Bioaccessibility is the quantity of soluble minerals released during simulated in vitro or in vivo gastrointestinal digestion, relative to the total mineral concentration in the food (Fairweather-Tait et al., 2007). By application of INFOGEST—a consensus, widely accepted simulated digestion protocol (Brodkorb et al., 2019)—we found that white-flour fractions of the VIT-NAS line released significantly more iron and zinc during the gastric and duodenal digestion phases relative to control flour from a null transformant line (Table 2). In the duodenal digestion phase, iron and zinc from control white flour was largely precipitated, but more than double the amount of each mineral remains soluble in the digestate of VIT-NAS white flour (Table 2). A similar improvement of mineral bioaccessibility was observed using a simulated gastrointestinal digestion procedure developed for iron uptake studies in Caco-2 cells (Glahn et al., 1998). Roller-milled white flour from the B71 line released 3-fold more iron and 1.3-fold more zinc compared to flour from a null transformant (Table 3). Taken together, we see that the dramatically increased levels of NA in the VIT-NAS lines correspond with enhanced iron and zinc bioaccessibility.

**Table 2:** Iron and zinc concentrations in gastrointestinal digests

Mineral	Sample		Gastric Digest End Phase			Duodenum Digest End Phase		
		Total (µg)	Soluble, reagent derived	Supernatant	Pellet	Soluble, reagent derived	Supernatant	Pellet
Fe	Control (A1)	1.64	0.03	0.43 ± 0.02	1.26 ± 0.09	0.63	0.57 ± 0.11	2.23 ± 0.02
	VIT-NAS (B71)	3.01		0.52 ± 0.03^*^	2.49 ± 0.20		1.09 ± 0.10^***^	3.20 ± 0.01
Zn	Control (A1)	2.19	0.10	2.07 ± 0.10	0.28 ± 0.08	3.06	1.63 ± 0.14	4.14 ± 0.13
	VIT-NAS (B71)	4.13		3.78 ± 0.08^**^	0.47 ± 0.56		4.45 ± 0.25^***^	3.54 ± 0.09

Amount of mineral in the soluble (supernatant) and insoluble (pellet) fractions at the end point of simulated digests using the INFOGEST method (Brodkorb et al., 2019). Values are the average of three replicates ±sd. Student’s *t* test between control and VIT-NAS for the supernatant fractions;

* *P* < 0.05,

** *P* < 0.01,

*** *P* < 0.001.

**Table 3:** Iron and zinc concentrations in gastrointestinal digests

Mineral	Sample	Total (µM)	Released (µM)
Fe	Control (22-15)	6.30	6.22 ± 0.70
	VIT-NAS (B71)	31.53	20.50 ± 0.87^***^
Zn	Control (22-15)	10.80	5.01 ± 0.47
	VIT-NAS (B71)	15.96	8.03 ± 0.25^**^

Concentration of mineral released during simulated gastrointestinal digestion following the method in (Glahn et al., 1998). Values are the average of three replicates ±sd. Student’s *t* test between control and VIT-NAS for the released mineral;

** *P* < 0.01,

*** *P* < 0.001.

## Discussion

Stacking transgenes to improve a specific trait in crops can also provide information on the physiological role of each gene and its contribution to this trait. When combining the gene cassettes for endosperm-specific *TaVIT2* and constitutive *OsNAS2* expression, previously reported changes in mineral content and distribution associated with each transgene were observed, but there were also a number of unexpected results. First, total grain iron was not increased in three out of four VIT-NAS lines (Figure 3B), in contrast to the 30%–50% increase reported in studies overexpressing *OsNAS2* alone (Singh et al., 2017; Beasley et al., 2019). Possibly, the transgene expression of *TaVIT2* counteracts the effect of *OsNAS2* regarding iron translocation into the grain. It should also be noted that previous studies used the variety Bobwhite, whereas we used Fielder and Gladius. Nevertheless, the marked redistribution of iron from the bran fractions to the white flour (starchy endosperm) is similar in lines expressing *HMWG::TaVIT2* alone or in combination with *OsNAS2*, which provides a great nutritional benefit to white flour.

A second unexpected finding was that the *TaVIT2* transgene, placed under the control of an endosperm-specific promoter, was also highly expressed in the leaves in wheat lines that had multiple T-DNA insertions (Figure 1B). The *HMWG* promoter upstream of *TaVIT2-D* is well characterized as an endosperm-specific promoter (Lamacchia et al., 2001), and indeed the A10 line, with a single-copy insertion of the T-DNA, showed the expected expression pattern. We hypothesize that in multi-copy insertion lines the 35S promoter of the HYG resistance marker acts as an enhancer for *TaVIT2-D* expression as noted previously in Arabidopsis (*Arabidopsis thaliana*) studies (Yoo et al., 2005 and references therein). Expression data from the expVIP database (www.wheat-expression.com) indicate that normally there is 3.5-fold higher expression of the *TaVIT2* homeologs in the leaves compared to grain (Connorton et al., 2017), in broad agreement with our RT–qPCR analysis of 2.6-fold higher expression in leaves (Supplemental Figure S6). In the B14 line, the leaf-to-grain ratio of total *TaVIT2* expression (endogenous plus transgene) was increased to 5.3. Remarkably, iron was not found to accumulate in the leaves of the B12 and B14 lines (Figure 3D). This suggests that certain physiological conditions need to be in place for vacuolar iron accumulation, including sufficient intracellular iron flux and low expression of genes for iron export from the vacuole, such as NRAMP3 and NRAMP4 (NATURAL RESISTANCE-ASSOCIATED MACROPHAGE PROTEIN). For the same reasons, it is thought that iron does not accumulate in the “lobes” of TaVIT2 grain, despite high expression of the *TaVIT2* transgene as shown by in-situ hybridization, because this part of the endosperm is not involved in translocating iron to the embryo as shown by ^57^Fe labeling (Sheraz et al., 2021). In contrast to some insight of how *VIT* overexpression affects iron distribution, we are far from understanding how constitutive *NAS* overexpression affects iron and zinc homeostasis in different plant organs. A study using the isotope ^55^Fe in Arabidopsis phagocytosis mutants has been very insightful in demonstrating the role of phagocytosis in iron recycling and remobilization to the seeds (Pottier et al., 2019), and isotope labeling could be applied to *NAS*-overexpressing plants.

A third unexpected finding is that the constitutive expression of *OsNAS2* influenced grain iron distribution that was altered by *TaVIT2* expression, providing additional insights into the poorly understood process of iron distribution during grain development. Like the TaVIT2 line, the VIT-NAS lines accumulated iron in vacuolar globules within cells of the starchy endosperm that are located between the vascular bundle and embryo (Figure 4B). However, unlike the TaVIT2 line, the VIT-NAS line also retained iron within the aleurone layer, similar to that observed in the null control (Figure 4B). Isotope labeling studies combined with NanoSIMS showed that iron is trapped in endosperm vacuoles during the nutrient-filling stage of grain development in TaVIT2 lines (Sheraz et al., 2021). We speculate that the retention of iron within the endosperm prevents iron from completing its movement to the aleurone layer. In contrast, the overexpression of *OsNAS2* in the VIT-NAS lines seems to maintain the mobilization of iron into the aleurone layer despite the overexpression of *TaVIT2* (Figure 4B).

Crucially, the absolute levels of zinc of at least 45 µg g^−1^ in the VIT-NAS lines are above the biofortification target of 38 µg g^−1^ (dry weight) set for whole wheat flour by HarvestPlus (Bouis et al., 2011). The target for iron is 59 µg g^−1^, but this number assumes only 5% bioavailability in wholemeal flour. Depending on the cultural preference for consuming white or wholemeal flour, and the expected improvement in iron bioavailability, the iron concentrations of 25 µg g^−1^ and 30 µg g^−1^ in white and wholemeal flour, respectively, may be sufficient to achieve 30% of the estimated average requirement of iron in the diet. Recent studies have shown that the NA concentration of cereals (rice and wheat) is a major determinant of iron bioavailability (Eagling et al., 2014; Beasley et al., 2019, 2021). NA conjugates of iron, and possibly also zinc, are thought to be taken up by intestinal cells using the proton-coupled amino acid transporter 1, a separate route from Fe^2+^ uptake by the divalent metal transporter 1 (Murata et al., 2021). NA is apparently not degraded by baking (Beasley et al., 2021), but the topic of heat stability of NA within food matrices awaits further study.

INFOGEST is fast emerging as the model digestion protocol to assess the bioaccessibility of multiple micronutrients including iron. The Glahn protocol (Glahn et al., 1998) is currently used as a standard in the iron nutrition community for assessing iron release and subsequent absorption by means of ferritin production in Caco-2 cell culture, which has a strong positive association with results from human trials (Fairweather-Tait et al., 2007). While the Glahn protocol is facile, INFOGEST provides a robust framework for controlling many parameters including enzyme activity. In this study, improved iron release associated with the VIT-NAS flour was reflected in both digestion protocols. Phytate concentration was not significantly different between control and VIT-NAS flours (Supplemental Figure S4). For this reason, we argue that the enhanced mineral bioaccessibility seen in VIT-NAS white flour digests is a consequence of iron or zinc bound to NA which may exert preferential cation chelation compared with phytic acid. This hypothesis will be investigated by HPLC-ICP-MS in the near future.

The ultimate aim is for biofortified wheat to be grown by farmers in different parts of the world and for it to be incorporated into human diets. We found no evidence of detrimental growth effects caused by the introduction of the VIT-NAS construct (Figure 2; Supplemental Figures S1 and S2). This will need to be further studied in field trials, but the initial data suggest that the VIT-NAS construct does not affect key agronomic traits and may thus be acceptable to farmers. It will also be important to confirm that the increased nutrient levels and bioaccessibility identified in these glasshouse trials can be replicated in the field. Promisingly, field trials for wheat with constitutive *OsNAS2* expression lines have been successful in this respect (Beasley et al., 2021). Importantly, we show the effect of the VIT-NAS construct in two distinct wheat cultivars, Fielder and Gladius. In particular, cv. Gladius is widely grown by farmers in Australia and is known for its useful agronomic traits such as drought tolerance. The success of the VIT-NAS construct in cv. Gladius, improving micronutrient levels with no observed impact on measured plant growth traits, indicates that it could be effectively incorporated into other elite wheat varieties. A further consideration when taking a genetic-modification approach for biofortification is the regulatory landscape governing the use of such lines. It may be beneficial to develop a version of the VIT-NAS construct which uses only wheat-derived genetic sequence within the T-DNA. Research into the most appropriate wheat *NAS* gene would be required, as would the replacement of the selection marker genes with alternatives.

In conclusion, the stacking of *HMWG::TaVITD-2* and *UBI1::OsNAS2* combines the effects of each transgene but does not impact plant growth. However, our study also revealed physiological and biotechnological aspects to be addressed in the future, such as the role of NA in translocating iron to the aleurone cells of the grain; and why elevated NA has a larger effect on increasing total zinc than on iron in grains. Moreover, it will be interesting to investigate to what extent iron and zinc speciation is changed in VIT-NAS flours, mostly with respect to NA and phytate complexes, and how this correlates with mineral bioavailability. Field trials are in progress to confirm the results and to bulk up material for making wheat products for nutritional studies.

## Materials and methods

### Vector construction and wheat transformation

The VIT-NAS construct was generated by inserting the *HMWG::TaVIT2-D* cassette (Connorton et al., 2017) between the T-DNA right border (RB) and *ZmUBI1*::*OsNAS2* cassette (Beasley et al., 2019) in a modified pMDC32 vector backbone (Curtis and Grossniklaus, 2003), see Figure 1A. The *HMWG::TaVIT2-D* sequence was inserted by In-Fusion cloning (Takara Bio, Shiga, Japan) in the HindIII restriction site upstream of *ZmUBI1::OsNAS2*, using primers JC172 and JC173 (Supplemental Table S1). Thus, the *TaVIT2-D* gene (*TraesCS5B02G202100*) is under the control of the *HMWG*-*D1* promoter (Lamacchia et al., 2001), resulting in ectopic expression of *TaVIT2-D* in the grain endosperm (Connorton et al., 2017). The *OsNAS2* gene (*Os03g0307200*) is under transcriptional control of the maize ubiquitin promoter *ZmUBI1* for constitutive expression (Christensen and Quail, 1996). The T-DNA also contains the plant-specific hygromycin resistance marker (Curtis and Grossniklaus, 2003). The *TaVIT2* and *OsNAS2* genes were terminated by the nopaline synthase terminator (*nosT*), while the hygromycin resistance gene was terminated by the CaMV 3′-untranslated region. See Supplemental Figure S5 for a detailed map of the vector construct.

The transformation of bread wheat (*T. aestivum* L.) cultivar Fielder was carried out at the John Innes Centre, following the protocol detailed in (Hayta et al., 2019). A zero-copy null transformant, referred to here as A1, was used as a control. At each generation, copy number analysis was carried out by iDNA Genetics (Norwich, UK) using a Taqman probe against the hygromycin resistance gene (Hayta et al., 2019). The grain from the homozygous T_3_ generation was used for the micronutrient content and NA analyses reported here. The TaVIT2 line used as a control has been described in Connorton et al. (2017) and Sheraz et al. (2021).

The transformation of wheat cultivar Gladius was carried out at the University of Adelaide following the protocol details in (Ishida et al., 2015). Copy number analysis was carried out on the T_0_ plants by real-time quantitative PCR (qPCR) using primers specific to the VIT-NAS construct (Supplemental Table S1). Individual T_0_ plants which contained single-copy inserts (denoted with a “-T” ending) were selected for further characterization in the T_2_ generation, alongside sibling null segregant lines (denoted with the “-N” ending).

### Plant growth

For all cv. Fielder experiments, seeds were pre-germinated for 48 h at 4°C on moist filter paper. They were then sown into P96 trays containing peat-based soil (85% (v/v) fine peat, 15% (v/v) horticultural grit) without NPK or mineral fertilizers. At 21 days, the plants were transplanted into 1-L individual pots containing Petersfield Cereal Mix (Petersfield, Leicester, UK). The plants were arranged in a randomized block design and grown in standard glasshouse conditions with 16-h:8-h light:dark cycles.

For all cv. Gladius experiments, seeds were pre-germinated for 48 h at 4°C, after which they were germinated in P56 plug trays containing soil supplemented by 4.5 g L^−1^ Osmocote (Scotts Australia). After 2 weeks, plants were transplanted into 1-L pots containing the same soil. The plants were grown in a randomized block design, initially at a constant temperature of 24°C during the day, and 18°C at night. After transplanting to individual pots, the plants were moved to an accelerated “speed breeding” growth condition (Watson et al., 2018).

### Plant phenotyping

Plant phenotyping data from the cv. Fielder plants were obtained at harvest. Plant height was measured from the soil to the tip of the highest spike, excluding awns. The entire above-ground plant was harvested and dried at 35°C for 1 week to obtain the total above-ground dry weight. Harvest index was calculated as the ratio of the total plant grain yield (g) to the total above-ground dry weight (g). After threshing, grain yield per plant was calculated and the thousand grain weight and grain area, width, and length were obtained using the MARVIN seed analyzer (GTA Sensorik GmbH). Plant phenotyping data from the cv. Gladius plants were obtained in the same manner, with the exception that plant material was dried at 45°C for 72 h to obtain the total above-ground dry weight.

### RT–qPCR

Leaf and grain tissues were sampled from individual T_3_ VIT-NAS plants (cv. Fielder) at 21 days postanthesis and snap frozen in liquid N_2_. The snap-frozen tissue was then ground in liquid N_2_ to a fine powder and RNAs were extracted using TRIzol Reagent (Thermo Fisher Scientific, Waltham, MA, USA). RNA concentration and purity were checked using a Nanodrop instrument (Thermo Scientific). DNase treatment was carried out using RQ1 RNase-Free DNase (Promega, Madison, WI, USA) before cDNAs were synthesized using the Invitrogen M-MLV reverse transcriptase.

Primers specific to the gene sequences introduced within the VIT-NAS construct were used, while previously published primers were used for the internal control *TaACTIN* (Supplemental Table S1) (Uauy et al., 2006). Primer efficiencies were calculated using pooled grain cDNA from lines B4, B14, B12, and B71. RT–qPCR reactions were performed using the LightCycler 480 SYBR Green I Master Mix with a LightCycler 480 instrument (Roche Applied Science, UK) under the following conditions: 5 min at 95°C; 45 cycles of 10 s at 95°C, 15 s at 60°C, 30 s at 72°C; followed by a dissociation curve from 60°C to 95°C to determine primer specificity. In all cases, three technical replicates were carried out per sample and the construct-specific expression of *TaVIT2-D* and *OsNAS2* was recorded relative to *TaACTIN*.

### Flour production

For cv. Fielder, grains were hydrated to ∼12% moisture content and milled with an IKA Tube Mill 100 for 2 min at 25,000 rpm. The resulting wholemeal flour was passed through a 150-µm Nylon mesh to separate a crude white-flour fraction from the bran. The wholemeal flour and the white-flour fraction were retained for downstream analysis.

For cv. Gladius, grains were washed in a 0.1% (v/v) Tween-20 solution, rinsed in distilled water, and dried at 60°C for 48 h before milling using an IKA Tube Mill for 30 s at 20,000 RPM. The wholemeal flour was then passed through a 200-µm Nylon mesh to obtain the white-flour fraction.

For roller-milled flour fractions, grain was hydrated to ∼15% moisture content and milled using a Chopin CD1 laboratory mill. The pooled white flour for bioaccessibility assays consisted of 9.5 g of Break 1, 11.5 g of Reduction 1, 1.5 g of Break 2, and 2 g of Reduction 2 for the control flour of the 22-15 null transformant line; 9.8 g of Break 1, 11.5 g of Reduction 1, 1.5 g of Break 2, and 2 g of Reduction 2 for the B71 line.

### Elemental analysis

For cv. Fielder samples, flour was dried overnight at 65°C. Flour samples (0.1 g) were digested in 55% (v/v) nitric acid and 6% (v/v) hydrogen peroxide at 95°C for 17 h. The acid-digested samples were 5× diluted with ultra-pure analytical-grade water. The samples were analyzed using ICP-OES (PlasmaQuant PQ 9000 Elite, Jena Analytik, Germany). Calibration was performed using 0, 0.125, 0.25, 0.5, 1, 2, 3, 4, and 5 µg g^−1^ standards of P and Mg and 0, 0.025, 0.05, 0.1, 0.2, 0.4, 0.6, 0.8, and 1 µg g^−1^ standards of Mn, Fe, and Zn. Rhodium, to a final concentration of 0.1 µg g^−1^, was used as an internal standard. Hard red spring wheat reference material (National Research Council Canada) was treated in the same manner as the experimental samples and included in every run of 50 samples.

For cv. Gladius samples, the white-flour fraction was analyzed using ICP-OES following standard procedures at the Trace Analysis for Chemical, Earth, and Environmental Sciences (TrACEES) platform (Parkville, University of Melbourne). The 1567b wheat flour was used as the standard reference material (National Institutes of Standards and Technology, MD, USA).

### NA quantification

The concentration of NA was measured in water-based extracts using liquid chromatography–mass spectrometry (LC–MS), following the method in Banakar et al. (2017) with minor modifications. Approximately 0.5 g of wholemeal flour was mixed with 282 µL of milliQ water and 18 µL of 1 mM *N*^ε^-nicotinoyl-L-lysine (see below) which was added as internal standard. The mixture was homogenized for 5 min at 1,000 rpm with a motorized pestle tissue grinder and centrifuged at 4°C for 15 min at 15,000*g* to recover the supernatant. The pellet was extracted another 4 times with 300 µL water and the 5 supernatants pooled, then filtered through 3 kDa filters (Amicon Ultra) at 4°C at 15,000*g* for 60 min. The extracts and 1 µM, 10 µM, 100 µM, and 200 µM standard solutions of NA (US Biological Life Sciences) each containing 83 mM *N*^ε^-nicotinoyl-L-lysine were lyophilized overnight. The residues were dissolved in 20 µL of milliQ water, 10 µL 50-mM EDTA, and 30 µL of mobile phase A (1:10 ratio of 10-mM ammonium acetate to acetonitrile, pH 7.1) before being filtered through 0.45-µm polyvinylidene fluoride ultrafree-MC centrifugal filters (Durapore, Merck) for 10 min at 12,000*g*. The samples were then analyzed by LC–MS (Xevo TQ-S, Waters). The separation was performed using a Waters Acquity Ultra Performance LC system and a µLC column (SeQuant ZIC-HILIC, 150 × 1 mm internal diameter, 5 µm, 200 Å) equipped with a guard column. The flow rate of the mobile phases was set to 0.15 mL min^−1^. The gradient program was set to 100% mobile phase A for 3 min; a linear gradient to 30% A and 70% B (8:2 ratio of 30-mM ammonium acetate to acetonitrile, pH 7.3) over 7 min; 30% A and 70% B for 7 min; a gradient to 100% A for 8 min; 100% A for 10 min. The total run time for each sample was 35 min, the injection volume was 5 µL and the autosampler temperature was 6°C, whilst the column was at room temperature. The liquid chromatography system was coupled to a time-of-flight-mass spectrometer with a negative electrospray source. The spray chamber conditions were set to a spray voltage of 1.5 kV, a desolvation temperature of 500°C, flow rates were 900 h^−1^ and 150 h^−1^ for the desolvation and cone gas, respectively, and the nebulizer pressure was set to 7.0 bar. The TargetLynx V4.1 (Waters Inc., Milford, MA, USA) software was used for quantification. The 302–186 *m/z* daughter transition was used to quantify NA and the 250–78 *m/z* daughter transition was used to quantify *N*^ε^-nicotinoyl-L-lysine.

### Bioaccessibility assays

Simulated digestion following the INFOGEST method was performed as described in Minekus et al. (2014) with minor modifications. Hand-milled white flour (0.2 g) was mixed with 600 µL of water to obtain a homogeneous bolus. For the oral phase, 640 µL of simulated salivary fluid was added to the bolus, 4 µL of 0.3 M CaCl_2_(H_2_O)_2_, and 156 µL of water. Human salivary amylase was omitted as oral starch digestion was not of interest. The mixture was incubated for 2 min at 37°C. For the gastric phase, the pH was adjusted to 3.0 using 0.5-M HCl, and 1.36 mL of simulated gastric fluid (as defined in Minekus et al. (2014)), 0.8 µL of 0.3 M CaCl_2_(H_2_O)_2_, and 96 µL of water was added. Pepsin (Merck P7012, pepsin from porcine gastric mucosa) was added to a final concentration of 2,000 U mL^−1^. Lipases were omitted owing to the low lipid content of the flours. Samples were incubated for 1 h at 37°C. For the duodenal phase, 1.68 mL of simulated intestinal fluid (46) was added to the gastric mixture, adjusting the pH to 7.0. To this was added 10-mM bovine bile salts (Merck B3883), 6.4 µL of 0.3 M CaCl_2_(H_2_O)_2_, 100-U mL^−1^ porcine trypsin (Merck T4799), 25-U mL^−1^ bovine chymotrypsin (Merck, Kenilworth, NJ, USA; C4129) and 200-U mL^−1^ porcine pancreatic α-amylase (A3176), was added. Pancreatic lipase and colipase were omitted. The intestinal phase was incubated at 37°C for 2 h.

At the end of each digestion phase, samples were centrifuged at 1,000*g* for 5 min. Supernatants were decanted for analysis of soluble minerals; pellets were resuspended in 1 mL of analytical grade water and also analyzed to obtain total values of iron and zinc for each sample. Gastric endpoint samples (2.5-mL supernatant) were acid mineralized in 16% (w/v) HNO_3_ and 1.7% (w/v) 0.2-mL H_2_O_2_ at 95°C for 16 h, then made up to 4 mL with ultrapure water. Duodenal endpoint samples (6.4-mL supernatant) were frozen in liquid nitrogen and lyophilized prior to elemental analysis as described for flour.

Simulated gastrointestinal digestion following the Glahn protocol was performed as described in Glahn et al. (1998). Pooled, roller-milled white-flour (1 g) samples were mixed with 10 mL of saline solution (140-mM NaCl and 5-mM KCl, pH 2.0) and adjusted to pH 2.0 with 1-M HCl. To this, 0.5 mL of pepsin (Sigma-P7000; 16 mg ml^−1^) was added. Samples were incubated at 37°C on a rocking platform (150 rpm) for 90 min. The pH of the samples was then adjusted to pH 5.5 using 1-M NaHCO_3_. Bile extract (Sigma-B8631; 8.5 mg mL^−1^) and pancreatin (Sigma-P1750; 1.4 mg L^−1^) were added at 2.5 mL, and the pH was adjusted again to pH 7.0. The solution was made up to 16 mL with saline solution (140-mM NaCl and 5-mM KCl, pH 7.0), and the samples were incubated at 37°C for 90 min. Samples were centrifuged at 1,000*g* for 5 min to separate the soluble mineral fraction and insoluble food pellet. For mineral quantification, 250 µL of supernatant was added to 14.75-mL HNO_3_ (17.5% w/v) containing 1 mg L^−1^ Yttrium (Merck Millipore) as an internal standard. Mineral content in the samples was analyzed using ICP-OES (Thermo Fisher iCAP 6300).

### Iron staining

Whole, mature wheat grains were cut with a platinum-coated razor blade and immersed in 2% (w/v) potassium ferrocyanide and 2% (v/v) HCl for 45 min, then washed with water and photographed. Grain cross sections (1 µm) were prepared as previously described, mounted on glass slides, and stained for iron using the Perls’ method enhanced with diaminobenzidine (Sheraz et al., 2021). In brief, the slides were immersed in 2% (w/v) potassium ferrocyanide and 2% (v/v) HCl for 45 min. After washing with distilled water, samples were incubated in methanol containing 0.01 M NaN_3_, and 0.3% (w/v) H_2_O_2_ for 60 min. The slides were washed with 0.1 M phosphate buffer (pH 7.4) and incubated for 30 min with 1.8% (w/v) CoCl_2_.6H_2_O and 0.025% (w/v) diaminobenzidine in phosphate buffer. The slides were washed once with distilled water and left to air dry overnight and mounted with DPX mountant (Merck). The slides were imaged using a Leica DM6000 at 10× magnification.

### Statistical analysis

Graphs and statistical analyses were done in MS Excel or in R. Details regarding the number of replicates and the statistical tests are given in figure legends.

### Accession numbers

Sequence data from this article can be found in the GenBank data libraries under accession numbers *OsNAS2*, AP014959; *TaHMWG*, XM_044595098; TaVIT2*-D*, XM_044541451; *ZmUBI1*, MP579820.

## Supplemental data

The following materials are available in the online version of this article.

**Supplemental Figure S1**. The VIT-NAS construct does not affect plant growth in cv. Fielder.

**Supplemental Figure S2**. The VIT-NAS construct does not affect plant growth in cv. Gladius.

**Supplemental Figure S3**. Expression of the VIT-NAS construct in cv. Gladius results in increased iron and zinc in white flour.

**Supplemental Figure S4**. Phytate levels in the cv. Fielder VIT-NAS lines are not increased.

**Supplemental Figure S5**. T-DNA plasmid used for wheat transformation.

**Supplemental Figure S6**. Expression of TaVIT2-D in leaves and grain.

**Supplemental Table S1**. Primers used in this study.

## Supplementary Material

kiac499_Supplementary_Data

## Notes

J.M.C., A.A.T.J., P.A.S., C.U., and J.B. conceived the study and the overall research plans. S.A.H., J.M.C., N.I.M.N., R.M., Y.M.L.M., and M.F.A. designed and performed the experiments. All authors analyzed the data. S.A.H. and J.B. wrote the article with contributions from all authors.

The author responsible for distribution of materials integral to the findings presented in this article in accordance with the policy described in the Instructions for Authors (https://academic.oup.com/plphys/pages/general-instructions) is Janneke Balk (Janneke.balk@jic.ac.uk).
