# Supplementary material for: A two-gene strategy increases iron and zinc concentrations in wheat flour, improving mineral bioaccessibility
Source: Plant Physiol. 2022 Oct 29;191(1):528–41. doi: 10.1093/plphys/kiac499 (PMC9806615; doi:10.1093/plphys/kiac499)
Supplement: kiac499_Supplementary_Data [file kiac499_supplementary_data.pdf]

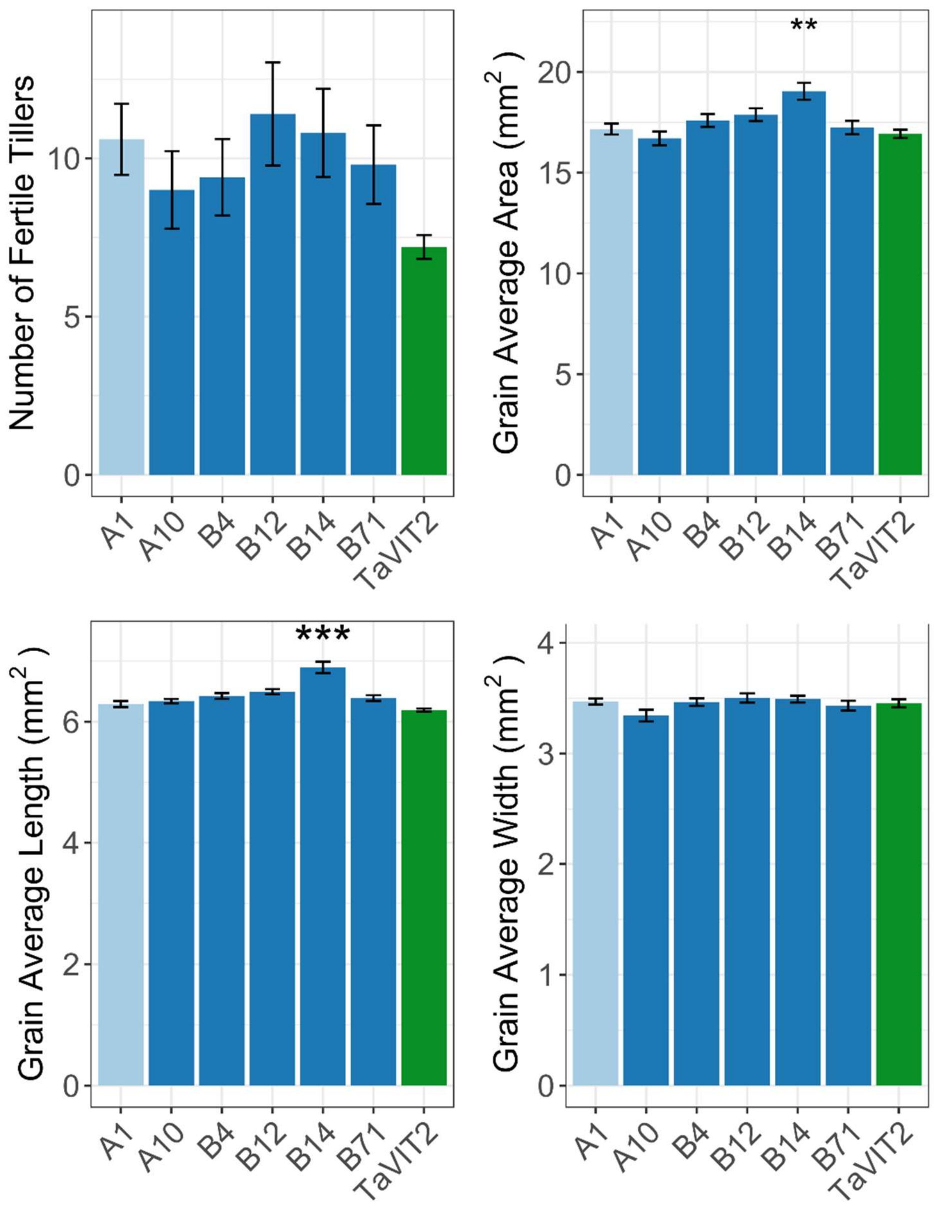

Supplemental Figure S1. The VIT-NAS construct does not affect plant growth in cv. Fielder.
Fertile tiller number and grain size parameters (area, length, and width) in the null transformant (A1; light blue), VIT-NAS (A10, B4, B12, B14, B71; dark blue), and TaVIT2 (green) lines (** $p < 0.01$, *** $p < 0.001$, Dunnett Test against A1). Error bars are the standard error of five biological replicates.

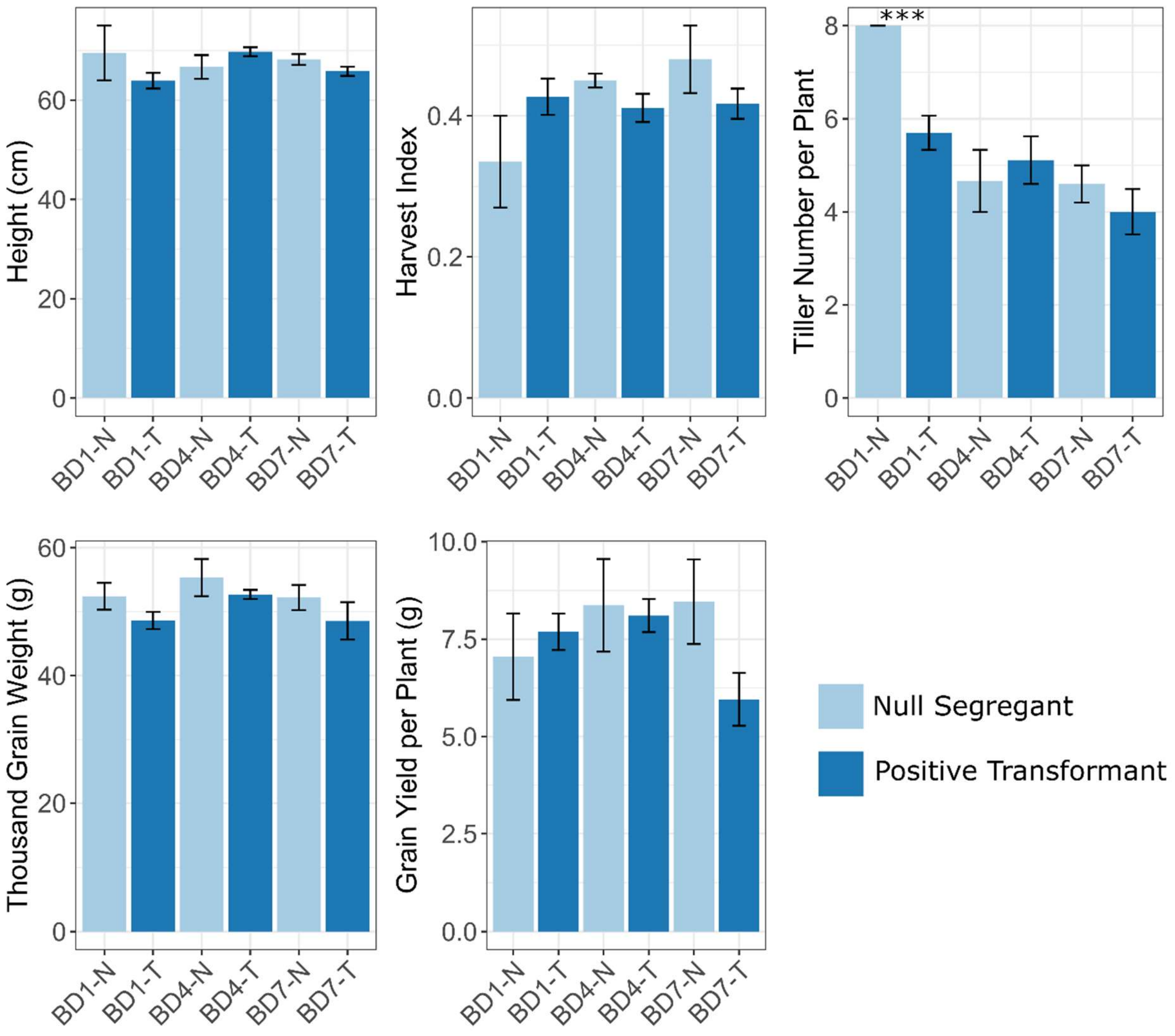

Supplemental Figure S2. The VIT-NAS construct does not affect plant growth in cv. Gladius.
Plant growth parameters including height, harvest index, tiller number, thousand grain weight, and grain yield per plant in three pairs of single-copy lines (dark blue, "-T") and their respective null segregant sibling (light blue, "-N). *** $p < 0.001$, Student's t-test. Error bars are the standard error of the biological replicates; n = 2 for BD1-N, 12 for BD1-T, 3 for BD4-N, 7 for BD4-T, 5 for BD7-N, and 7 for BD7-T.

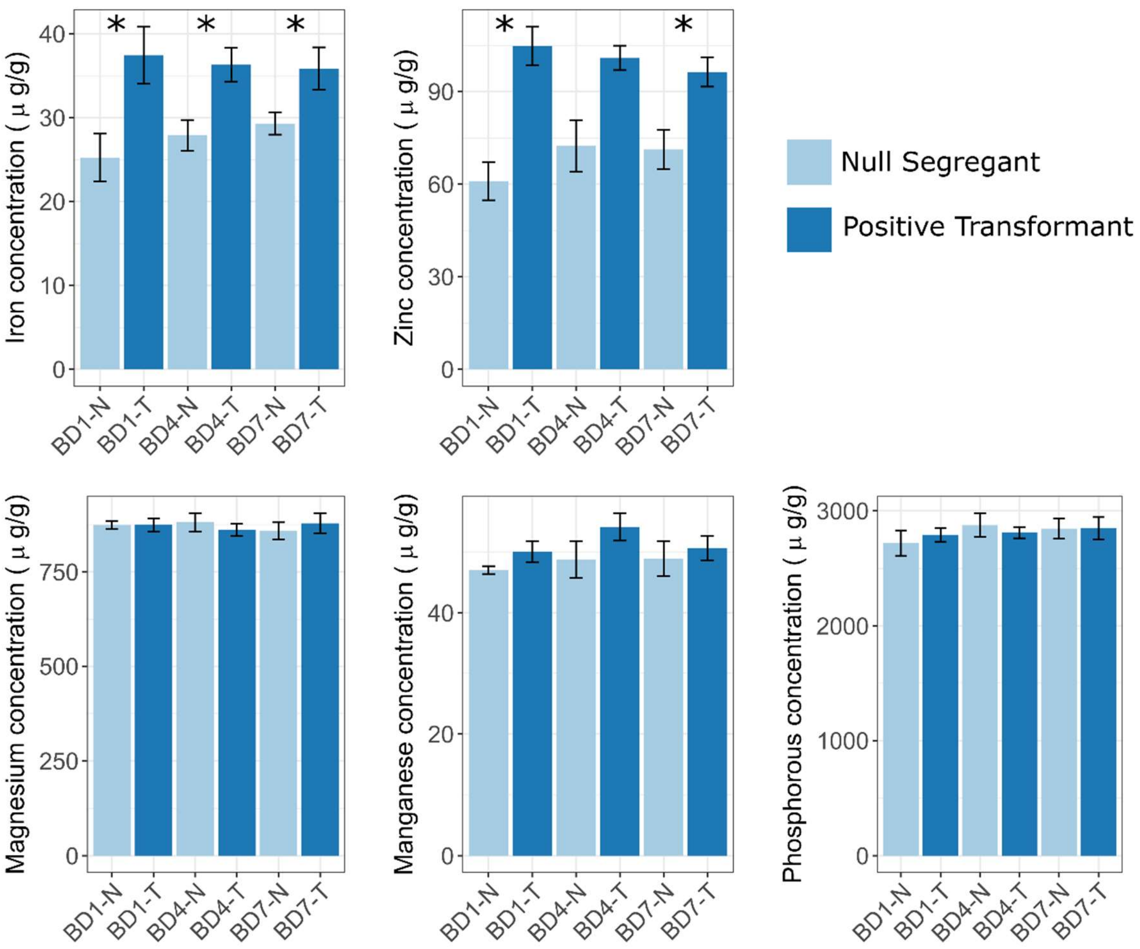

Supplemental Figure S3. Expression of the VIT-NAS construct in cv. Gladius results in increased iron and zinc in white flour. Micronutrient levels in hand-milled white flour for the cv. Gladius VIT-NAS lines, in three pairs of single-copy lines (dark blue, "-T") and their respective null segregant sibling (light blue, "-N). Error bars are the standard error of the biological replicates; n = 2 for BD1-N, 12 for BD1-T, 3 for BD4-N, 7 for BD4-T, 5 for BD7-N, and 7 for BD7-T. Student's t-test was carried out for each pair against the null segregant; *, $p < 0.05$, **, $p < 0.01$, ***, $p < 0.001$.

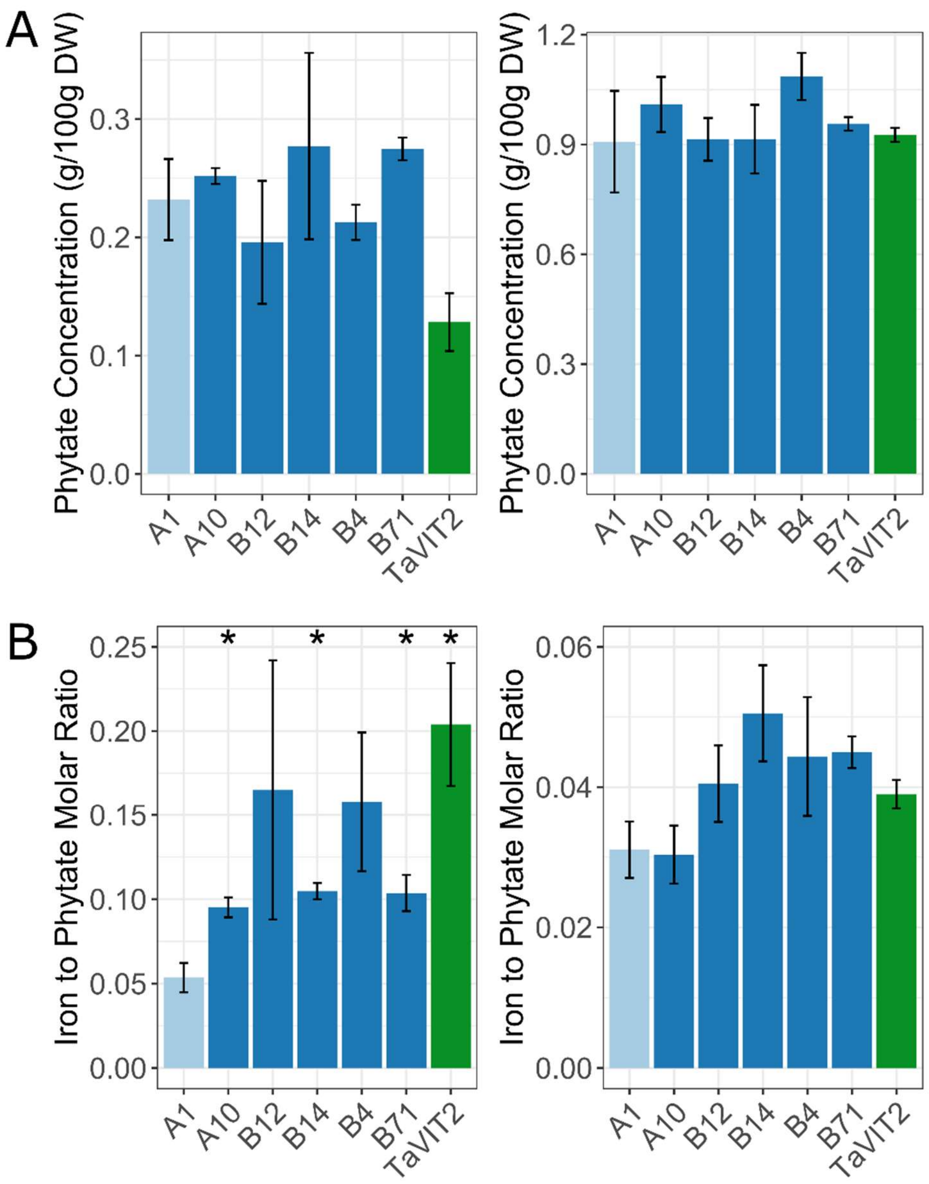

Supplemental Figure S4. Phytate levels in the cv. Fielder VIT-NAS lines are not increased.

A, Phytate levels were measured in the white (left) and wholemeal (right) flour from the null transformant (A1; light blue), VIT-NAS (A10, B4, B12, B14, B71; dark blue), and TaVIT2 (green) lines. B, The ratio of iron to phytate in the white (left) and wholemeal (right) flour for the null transformant (A1; light blue), VIT-NAS (A10, B4, B12, B14, B71; dark blue), and TaVIT2 (green) lines. Error bars are the standard error of 3 biological replicates. *, $p < 0.05$, **, $p < 0.01$, ***, $p < 0.001$; Student's t-test against the null (A1).

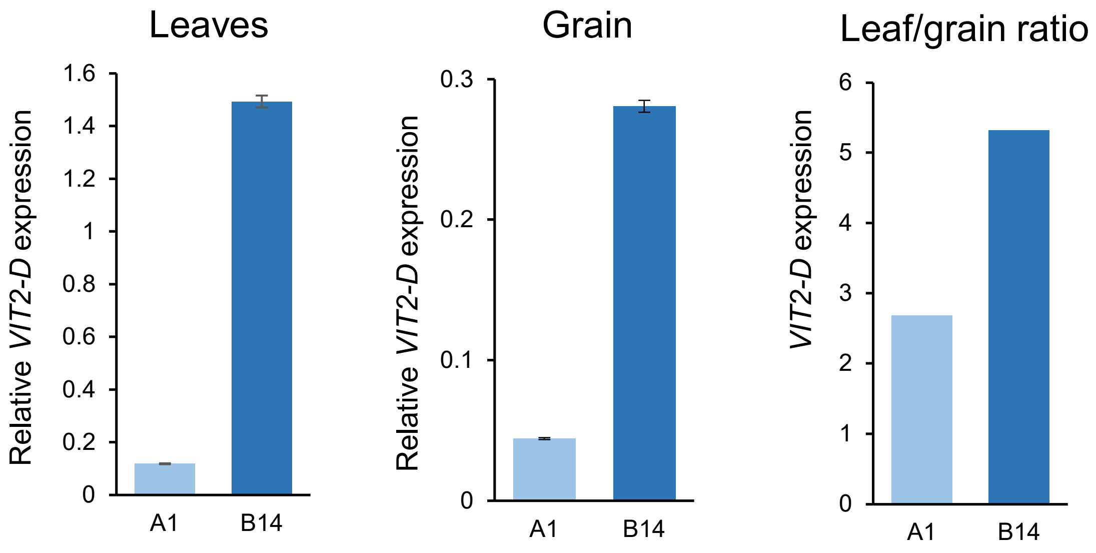

Supplemental Figure S6. Expression of *TaVIT2-D* in leaves and grain.

RT-qPCR expression of *TaVIT2-D* using primers ERJ09 and ERJ10 (see Table S1) which amplify both the endogenous and transgene copy. cDNA was prepared from flag leaf (left) and grain (right) tissue at 21 days post-anthesis from null transformant A1 and transgenic B14 lines. Expression levels were calculated relative to *TaACTIN*. Error bars represent the standard error of three biological replicates for each line.

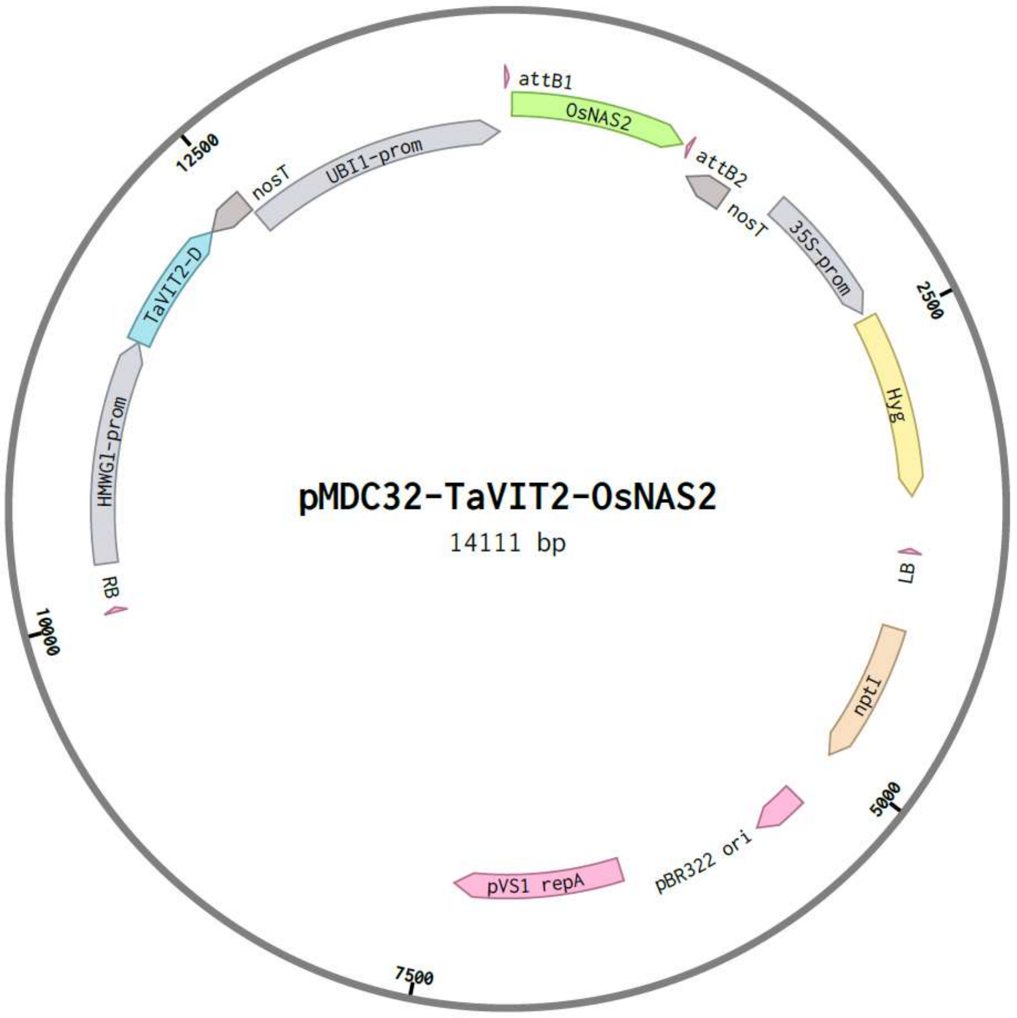

Supplemental Figure S5. **T-DNA plasmid used for wheat transformation.**

RB, Right Border of T-DNA; *HMWG1-prom*, promoter sequence of the High Molecular Weight *GLUTENIN-D1* gene; *TaVIT2-D*, open reading frame of the *VACUOLAR IRON TRANSPORTER 2*, D homoeolog (TraesCS5B02G202100) from wheat; *nosT*, nopaline synthase terminator; *UBI-prom*, promoter sequence of the maize *UBIQUITIN1* gene; attB1 and attB2, sequence elements (25 nt) for Gateway cloning; *OsNAS2*, open reading frame of the *NICOTIANAMINE SYNTHASE 2* gene (Os03g0307200) from rice; *35S-prom*, promoter sequence of the Cauliflower Mosaic Virus; *Hyg*, open reading frame of the plant selectable marker hygromycin phosphotransferase; LB, Left Border of T-DNA; *nptI*, open reading frame of the bacterial selectable marker neomycin phosphotransferase; pBR322 ori, origin of replication for plasmids in *Escherichia coli; pVS repA,* one of the genes for replication and stability of the plasmid in *Agrobacterium tumefaciens* (not all genes of the replicon are depicted).

Supplemental Table S1. Primers used in this study.

Primer Name	Purpose	Sequence	Efficiency	Source
JC172	Cloning	GGCCAGTGCCAAGCTCGAAATATGCAACATAATTTCC	NA	This paper
JC173	Cloning	GCAGGCATGCAAGCTCAGTAACATAGATGACACCG	NA	This paper
OsNAS_F	RT-qPCR	GTTCCAGAAGGCGGAAGAGT	113%	This paper
OsNAS_R	RT-qPCR	AACGATCGGGGAAATTCG		
TaVIT_F	RT-qPCR	AGCGCCATGATGACCTCC	110%	This paper
TaVIT_R	RT-qPCR	CGGCAACAGGATTCAATCTTAAG		
ERJ09	RT-qPCR	CTCCCCCTACATGTTCGT		Connorton et al. 2017
ERJ10	RT-qPCR	CCCTTGACGTAGCCGAA		
TaACTIN_F	RT-qPCR	ACCTTCAGTTGCCCAGCAAT	99%	Uauy et al. 2006
TaACTIN_R	RT-qPCR	CAGAGTCGAGCACAATACCAGTTG		
Gladius_F	qPCR	TTTCTTCGGCTACGTCAAGG	NA	This paper
Gladius_R	qPCR	AAGACCGGCAACAGGATTC		
